# Supplementary material for: Diversity of Lacewings (Neuroptera) in an Altitudinal Gradient of the Tacaná Volcano, Southern Mexico
Source: Insects. 2022 Jul 19;13(7):652. doi: 10.3390/insects13070652 (PMC9315885; doi:10.3390/insects13070652)
Supplement: Supplementary file 1 [file insects-13-00652-s001.zip › insects-1783435-supplementary.pdf]

Table S1. Characteristics of the sampling sites along the altitudinal gradient of the Tacaná Volcano, Chiapas, Mexico.

Municipality	Locality	Coordinates	Altitude	Dominant vegetation	Sampled area
Cacahoatán	Finca Alianza	15°02'29.22"N, 92°10'13.14"W	661m-774m	Evergreen tropical forest and coffee plants	1.5 km^2
Cacahoatán	Ejido El Águila	15°05'33.24"N, 92°10'50.64"W	1050m-1393m	Cloud forest and coffee plants	1.9 km^2
Cacahoatán	Ejido Benito Juárez El Plan	15°05'27.18"N, 92°08'51.06"W	1406m-1767m	Cloud forest and coffee plants	2 km^2
Unión Juárez	Cantón Chiquihuites	15°05'43.74"N, 92°05'57.6"W	2057m-2460m	Cloud- Oak forest	2 km^2
Unión Juárez	Volcán Tacaná, Parador Papales-La Cabaña	15°06'56.46"N, 92°05'54.72"W	2884m-3246m	Pine-Oak forest	1.9 km^2
